# A Comparison of Treatments and Outcomes for Medullary versus Nonmedullary Colon Cancer: A Single Institutional Experience Showing a Worse Prognosis for Stage 3 Disease

**DOI:** 10.1155/2020/5783729

**Published:** 2020-03-27

**Authors:** A. Gupta, B. Protyniak, J. Dove, K. Chu, T. Erchinger, J. Bannon, J. Oxenberg

**Affiliations:** ^1^Geisinger Wyoming Valley, Wilkes-Barre, PA, USA; ^2^Geisinger Medical Center, Danville, PA, USA; ^3^Geisinger Community Medical Center, Scranton, PA, USA

## Abstract

**Background:**

Prior studies have shown a better prognosis with medullary colon cancer (MCC) compared to nonmedullary colon carcinomas (NMC); however, data are inconsistent and lacking the evaluation of treatments received. As we did not see similar survival outcomes, we aimed to retrospectively examine survival and receipt of treatment differences between MCC and NMC within the Geisinger Health System.

**Methods:**

The Cancer Registry was retrospectively reviewed for MCC and NMC from 2006 to 2017. Demographics and treatments were compared using *T*-test and chi-squared analyses, also comparing MCC to poorly differentiated (PD) or undifferentiated (UD) NMC. Overall survival was analyzed using Kaplan–Meier curves and log-rank tests.

**Results:**

33 MCC and 1775 NMC patients were identified and 31 (93.9%) MCC and 1433 (87.0%) NMC underwent resection. MCC were older (*p*=0.0002), had a higher Charlson Comorbidity Index (*p*=0.013) and were more likely right sided (*p*=0.013). Seven patients (22.6%) with MCC vs. 149 (10.4%) NMC underwent resection of contiguous organs. Overall median survival was significantly worse for MCC as compared to NMC (19.6 vs. 60.5 months, *p*=0.0002). Only stage 3 patients had a significantly worse median survival when compared to PD/UD NMC (9.6 vs. 47.2 months, *p* < 0.001). Contiguous organ resection and failure to receive chemotherapy were not found as contributing factors to decreased survival.

**Conclusion:**

Multiple previous studies showed a better prognosis for MCC compared to PD/UD NMC. We, however, found stage 3 patients had a worse prognosis which may be secondary to higher comorbidities, increased stage, and higher rate of UD.

## 1. Introduction

Medullary colon cancer (MCC) was differentiated from nonmedullary colon cancer (NMC) as a subtype of adenocarcinoma by the World Health Organization (WHO) and is described as poorly differentiated or undifferentiated tumors with solid sheets of cells lacking glandular formation [1]. This variant is commonly mismatch repair deficient with microsatellite instability and is also characterized by prominent eosinophilic cytoplasm, a pushing type border, marked lymphocytic infiltration, small nuclei, and prominent nucleoli [2].

While data regarding histologic differences between MCC versus NMC are prominent, literature regarding differences in treatment and survival for MCC compared to NMC with poor or undifferentiated pathology appear to be limited and conflicting. Prognosis for MCC is thought to be better compared to NMC since it rarely presents with nodal metastases or metastatic disease [2, 3]. Prior studies showed MCC patients have a better prognosis than undifferentiated (UD) NMC [3–5]. However, one study showed UD MCC typically present at stage 3 may actually have a worse prognosis than NMC of the same stage [3]. Since these tumors more commonly occur in elderly, it is possible that the inconsistent survival outcomes may be secondary to increased surgical morbidity or limited adjuvant treatments.

We aimed to retrospectively examine treatment and survival differences between MCC and NMC within the Geisinger Health System (GHS). We hypothesize that we would see a decreased survival for MCC patients due to our elderly population and comorbidities making adjuvant therapies and surgery less well tolerated.

## 2. Methods and Materials

A retrospective review of patients diagnosed with colon cancer from January 2006 to January 2017 within the GHS was performed via the Cancer Registry. International Review Board approval was obtained. Patients diagnosed with adenocarcinoma (including all subtypes) or medullary carcinoma of the colon were included. All tumor stages were included, and patients had to be evaluated and/or treated for their cancer within the GHS. Other tumor types were excluded as well as primary rectal and rectosigmoid cancers.

Demographics included age, sex, Charlson/Deyo Comorbidity Index, primary tumor location, and KRAS status. Treatments evaluated included observation, chemotherapy, radiation, and surgery as well as sequence of treatments. The type of surgical resection was also evaluated. Patients were separated by pathology where adenocarcinoma was compared to MCC. Demographic and treatment data for MCC and NMC were then compared by the pathologic stage using the AJCC 7^th^ edition [6]. MCC was then compared to PD and UD NMC. For each group, treatment differences were compared for procedure type, radiation administration, chemotherapy administration, and sequence of treatments. Overall survival was evaluated.

Categorical data were represented as frequency (percentage) and analyzed using the chi-squared test or Fisher exact tests. Continuous data were represented as mean ± standard deviation and analyzed using Student's *t*-test. Time to event data was represented using Kaplan–Meier curves and analyzed using log-rank tests. All statistical tests were two sided with a *p* value <0.05 considered to be statistically significant. Analyses were performed using SAS 9.4 (SAS Institute, Cary, NC).

## 3. Results

Thirty-three MCC and 1775 NMC patients were identified. 292 patients had PD or UD NMC. Patient characteristics found to be significantly different between MCC vs. NMC and MCC vs. PD/UD NMC are shown in Table 1. MCC patients were found to be older than all NMC (79.3 years old vs. 68.3 years, *p* < 0.001) and significantly older than PD/UD NMC (79.3 years old vs. 70.2 years, *p*=0.0002). MCC was more common in females where 78.8% of MCC were females vs. 49% in NMC (*p*=0.001) and 57.9% of PD/UD NMC (*p*=0.02). Charlson/Deyo Comorbidity Index was significantly higher for patients with MCC vs. NMC where it was greater than 1 in 42.5% of MCC vs. 24.4% of NMC (*p*=0.013) and 27.4% of PD/UD NMC (*p*=0.013 and *p*=0.032, respectively). Tumor location was more likely to be right sided with 72.7% of the MCC patients vs. 41.9% of NMC patients (*p*=0.0003) vs. 54.8% of PD/UD NMC (*p*=0.048). 9.8% of NMC patients and 10.3% of UD/PD had KRAS mutations. Patients with MCC were also more likely to have stage 2 (36.4%) or stage 3 (48.8%) disease (*p*=0.004).

A comparison of treatments received for MCC vs. NMC and MCC vs. PD/UD NMC can be seen in Table 2. Thirty-one patients (93.9%) with MCC and 1433 (87.0%) NMC underwent surgical resection. Seven patients (22.6%) with MCC vs. 149 (10.4%) NMC underwent resection of contiguous organs. No MCC patient received radiation, and 6 (18.2%) patients received chemotherapy only in the adjuvant setting. The 2 patients who did not undergo surgery did not have clinical staging available and did not undergo any treatments. When comparing all MCC to NMC PD/UD for all included patients, MCC were less likely to receive adjuvant chemotherapy (18.2% vs. 45.2%, *p*=0.008).

For each pathologic stage (stages 1–3), other treatments received for surgically resected MCC vs. NMC were then compared. No significant differences in treatments (radiation or chemotherapy) were seen for stages 1 and 2 MCC vs. NMC. Of all Stage 3 patients undergoing surgical resection, MCC were less likely to receive chemotherapy compared to NMC (25.0 % vs. 72.4 %, *p* = 0.004). Only 4 patients underwent chemotherapy with stage 3 MCC. Reasons for not receiving adjuvant chemotherapy in this group were patient refusal, comorbidities, or death. There was only 1 patient with MCC stage 4 disease, and therefore no conclusion could be drawn about differences in these patients.

**Figure 1:**
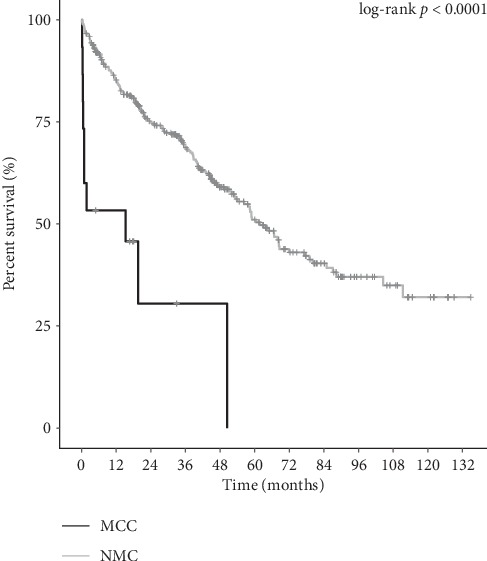
Kaplan–Meier curve stage 3 MCC vs. NMC.

**Figure 2:**
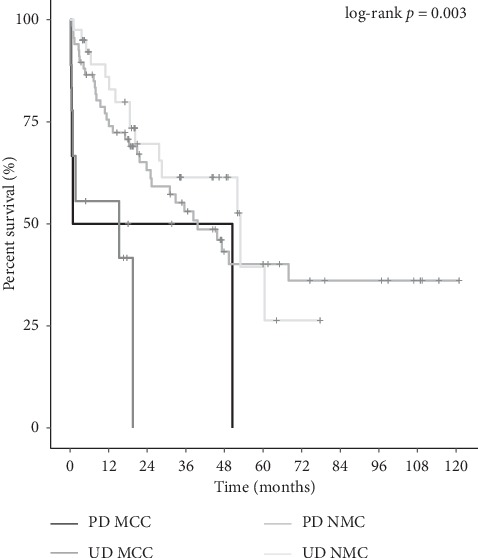
Kaplan–Meier curve stage 3 UD and PD MCC vs. UD and PD NMC.

When comparing the overall survival for all resected MCC vs. NMC by pathologic stage, only stage 3 patients were found to be significantly different (15.3 vs. 61.9 months, *p* < 0.0001). When comparing stage 3 MCC vs. PD/UD NMC, survival again was decreased for MCC (15.3 vs. 47.2 months, *p*=0.001) (Table 3). The Kaplan–Meier curve is displayed in Figure 1. Resected stage 3 PD and UD patients were then further separated and compared. Median overall survival continued to be worse for MCC vs. NMC for both groups. Overall survival was 25.7 months (*n* = 6) for PD MCC and 15.3 months for UD MCC (*n* = 9) vs. 39.6 months for PD NMC (*n* = 67), and 53 months for UD NMC (*n* = 40) (*p*=0.003) (Figure 2). While 7 patients (22.6%) with MCC vs. 149 patients (10.4%) with NMC underwent contiguous organ resections, survival comparisons showed MCC continued to have a worse survival even without contiguous organ involvement (19.6 months vs. 78.2 months, *p* < 0.0001). To determine if the worsened survival was secondary to difference in receipt of adjuvant chemotherapy, the resected stage 3 patients were compared. Median survival was not significantly different (*p*=0.06).

## 4. Discussion

Previous studies on MCC reported similar demographics to our findings, showing that MCC patients are older, predominantly female, and with right-sided tumors [3–5, 7]. However, our patient demographics do differ as compared to prior literature. Using the SEER database, Thirunavukarasu et al. identified 50 cases of MCC that more commonly presented as stage 2 disease as well as a higher rate of PD versus UD histology [3]. Wick et al. compared 68 patients with MCC with 35 PD “enteric” colorectal carcinomas and 15 neuroendocrine carcinomas and found MCC less commonly presents as stage 3 or 4 disease [7]. Our patient population was different, where the most common stage was stage 3 (48.5 % stage 3 vs. 36.4% stage 2) and UD pathology was also more common (36.4% PD vs. 60.6% UD).

Multiple prior studies also showed overall survival was favorable for MCC vs. NMS. Pyo et al. in 2016 found the overall survival rate of MCC higher than PD or UD NMC [5]. Wick et al. found that the MCC patients had a favorable prognosis with a 5-year mortality of 40% compared to 59% for the PD carcinomas [7]. Lastly, Thirunavukarasu et al. found that OS was improved compared to NMC, except for stage 3 patients with UD pathology [3]. In our series, all stage 3 MCC patients, despite differentiation, were found to have a worse prognosis than PD and UD NMC with a difference in over 46 months.

While Knox et al. compared MCC to other colorectal cancers with mismatch repair deficiencies and still found MCC to have a better prognosis, they also found patients with MCC may have a higher mortality at 30 days after resection [4]. After reviewing our institution's outcomes, our worse prognosis may be secondary to increased comorbidities, increased rate of UD pathology, and increased stage 3 tumors, thus making surgery less tolerated with increased postoperative mortality and higher risk of recurrence. MCC were less likely to get chemotherapy where only 4 of the 15 patients with stage 3 MCC who underwent surgery received chemotherapy. While the number of patients receiving chemotherapy is small and thus comparisons between these groups difficult, those that did not receive chemotherapy had a median overall survival of less than 1 month, highlighting a similar increased postoperative mortality. Resection of other contiguous organs did not lead to increased comorbidities.

In conclusion, our data showed that stage 3 MCC, with both PD and UD histology overall had a worse prognosis than NMC, contradicting some prior published literature. Our series indicate that surgery in this population may have increased risks for postoperative complications and death secondary to other comorbidities and a higher tumor stage with increased UD histology, increasing the risk of recurrence. Limitations of this study include a small sample size, retrospective nature, and a limited geographic population base. Because MCC is a relatively new diagnosis, there is ample room for studies in the future to elucidate the true prognosis of subtypes of MCC with a wider population base.

## Figures and Tables

**Table 1:** Patient characteristics for MCC vs. NMC and PD/UD NMC.

	MCC	NMC		MCC vs. NMC	MCC vs. PD/UD NMC
	Total (*n* = 33)	Total (*n* = 1775)	PD/UD (*n* = 292)	*p* value_a_	*p* value_b_
Age	79.3 ± 10.2	68.3 ± 13.3	70.2 ± 13.2	<0.0001	0.0002

Sex				0.001	0.020
Female	26 (78.8%)	869 (49%)	169 (57.9%)		
Male	7 (21.2%)	906 (51%)	123 (42.1%)		

Charlson/Deyo score				0.013	0.032
0	19 (57.6%)	1,342 (75.6%)	212 (72.6%)		
1	9 (27.3%)	309 (17.4%)	62 (21.2%)		
≥2	5 (15.2%)	124 (7%)	18 (6.2%)		

Location primary site				0.001	0.054
Ascending colon/cecum	24 (72.7%)	744 (41.9%)	160 (54.8%)		
Not ascending colon/cecum	9 (27.3%)	984 (55.4%)	130 (44.5%)		
Unknown/others	0	47 (2.7%)	2 (0.7%)		

Path stage				0.004	0.032
1	2 (6.1%)	287 (16.2%)	12 (4.1%)		
2	12 (36.4%)	386 (21.8%)	57 (19.5%)		
3	16 (48.8%)	391 (22%)	107 (36.6%)		
4	1 (3%)	258 (14.5%)	62 (21.2%)		
Unknown/others	2 (6.1%)	453 (25.5%)	54 (18.5%)		

Grade				<0.0001	0.001
Well differentiated	0	121 (6.8%)	0		
Moderately differ	0	1,113 (62.7%)	0		
Poorly differentiated	12 (36.4%)	198 (11.2%)	198 (67.8%)		
Undiffer/anaplastic	20 (60.6%)	94 (5.3%)	94 (32.2%)		
Unknown/others	1 (3%)	249 (14%)	0		

MCC, medullary colon cancer; NMC, nonmedullary colon cancer; PD, poorly differentiated; UD, undifferentiated.

**Table 2:** Treatments for MCC vs. NMC and MCC vs. PD/UD NMC.

	MCC	NMC	MCC vs. NMC	MCC vs. PD/UD NMC	
	Total (*n* = 33)	Total (*n* = 1775)	PD/UD (*n* = 292)	*p* value_a_	*p* value_b_
Surgery				0.424	0.553
No surgery	2 (6.1%)	227 (12.8%)	32 (11%)		
Surgery	31 (93.9%)	1544 (87%)	260 (89%)		
Unknown	0	4 (0.2%)	0		

Radiation				0.484	0.449
No	33 (100%)	1,749 (98.5%)	287 (98.3%)		
Yes	0	26 (1.5%)	5 (1.7%)		

Chemotherapy				0.529	0.008
None	27 (81.8%)	1,200 (67.6%)	157 (53.8%)		
Neoadjuvant	0	13 (0.7%)	3 (1%)		
Adjuvant	6 (18.2%)	547 (30.8%)	132 (45.2%)		
Neoadjuvant + adjuvant	0	12 (0.7%)	0		
Intraop with other therapies	0	3 (0.2%)	0		

MCC, medullary colon cancer; NMC, nonmedullary colon cancer; PD, poorly differentiated; UD, undifferentiated.

**Table 3:** Overall survival and follow-up of those surgically resected MCC vs. NMC.

	MCC	NMC	MCC vs. NMC	MCC vs. PD/UD NMC	
	Total	Total	PD/UD	*p* value	*p* value
Median overall survival, months (95% CI)					
Stage 1	Not reached	Not reached	Not reached	0.795	n/a
Stage 2	112.3 (14.1, NA)	97.8 (82.8, NA)	Not reached	0.301	0.283
Stage 3	15.3 (0.9, NA)	61.9 (54.1, 77.1)	47.2 (31.1, NA)	<0.0001	0.001
Stage 4	20.2 (NA, NA)	20.6 (16.3, 25.2)	10.4 (6.8, 16.5)	0.689	0.805

Median follow-up, months (95% CI)					
All patients	31.6 (17.7, NA)	57.7 (53.1, 61.2)	48.5 (45.9, 60.0)	0.015	0.031

Stage denotes pathologic stage. MCC, medullary colon cancer; NMC, nonmedullary colon cancer; PD, poorly differentiated; UD, undifferentiated.

## Data Availability

The data used to support the findings of this study are included within the article.
